# Isoperistaltic versus antiperistaltic uncut Roux-en-Y anastomosis after distal gastrectomy for gastric cancer: a propensity score matched analysis

**DOI:** 10.1186/s12893-020-00936-z

**Published:** 2020-11-07

**Authors:** Cui Hangtian, Huang Huabing, Luo Tianhang, Yin Xiaoyi, Fang Guoen

**Affiliations:** 1 Department of Gastrointestinal Surgery, Changhai Hospital, The Second Military Medical University, Shanghai, 200433 China; 2 Department of Gastroenterology, Changhai Hospital, The Second Military Medical University, Shanghai, 200433 China; 3 Department of General Surgery, Changhai Hospital, No.168 Changhai Road, Yangpu District, Shanghai, 200433 China

**Keywords:** Distal gastrectomy, Uncut Roux-en-Y anastomosis, Propensity score matching

## Abstract

**Background:**

The uncut Roux-en-y anastomosis (URYA) has some clinical advantages after distal gastrectomy (DG). Little evidence exists regarding the influence of peristalsis on this anastomosis. We aimed to evaluate short-term outcomes of isoperistaltic URYA (iso-URYA) comparing with antiperistaltic URYA (anti-URYA) after DG.

**Method:**

Patients who underwent URYA for gastric cancer (GC) between January 2016 and December 2018 were selected from Shanghai Changhai Hospital, Navy Medical University. Short-term outcomes were compared between iso-URYA group and anti-URYA group after 1:1 propensity score matching (PSM).

**Result:**

A total of 612 patients were selected. 392 patients underwent iso-URYA and 220 patients underwent anti-URYA. After PSM, 183 patients for each group were included in the final analysis. No differences were found between them in terms of short-term complications, nutritional status and quality of life 1 year after surgery. Endoscopic examination showed that anti-URYA group had more severe gastritis (P = 0.036). In addition, the recanalization rate was significantly higher when the afferent loop was blocked by stapler.

**Conclusion:**

The iso-URYA and anti-URYA group present similar results in short term outcomes. Ligation blocking afferent loop leads to lower recanalization rate.

## Background

Gastric cancer is the fifth common cancer globally and its incidence is increasing [1]. Surgical resection with radical lymphadenectomy is regarded as the basic treatment principle for patients with resectable locally advanced gastric cancer [2], while multiple variations have been detailed in the digestive reconstruction. In recent years, many studies indicated that uncut Roux-en-y anastomosis (URYA) after DG had some clinical advantages compared with Billroth I (BI), Billroth II (BII), BII with Braun and Roux-en-Y (RY) reconstruction [3–6]. The reason behind this lies in that URYA can maintain the integrity of the intestinal canal and further preserve myoneural continuity to eliminate Roux stasis syndrome through an occluded but not cut jejunogastric pathway [7, 8]. Despite this consensus, the operative details vary among surgeons, like ligation or stapler for luminal occlusion, site of occlusion, orientation of peristalsis. Actually, as far as we know, there are still no studies to evaluate functional effects of orientation of peristalsis on URYA.

In view of this uncertainty, we designed this retrospective study to evaluate the effects of iso- and anti-URYA after DG. Propensity score matching (PSM) was used with enough and appropriate subset of covariates to adjust the biased cohort. Short-term outcomes were compared between iso-URYA and anti-URYA after DG for gastric cancer.

## Method

### Study design

**Fig. 1:**
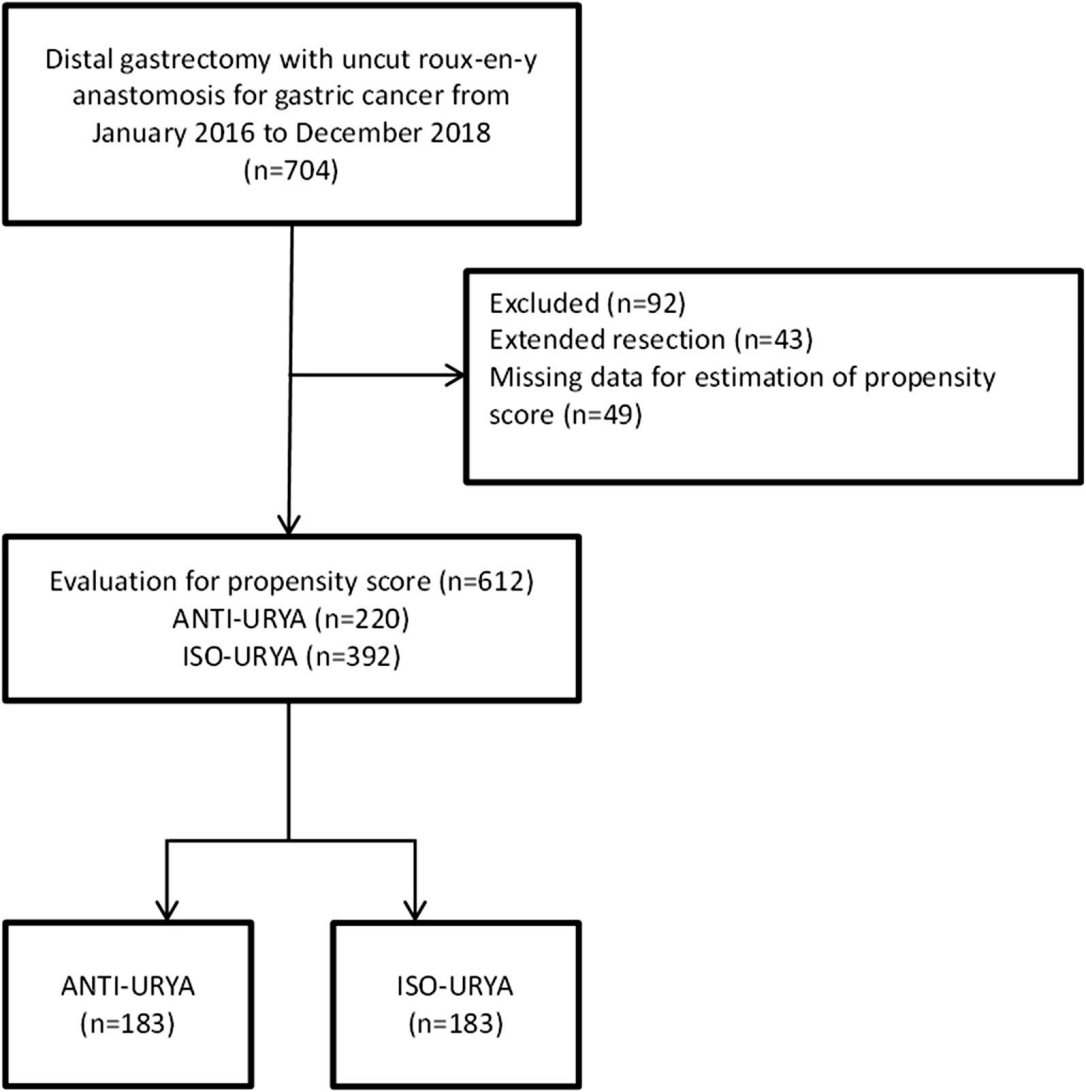
Flow chart

This retrospective study has been approved by the Research Ethics Committee of Changhai Hospital, Navy Medical University. The inform consent was exempted in this retrospective study. Demographic baseline and surgical variables of the patients who received DG at Changhai Hospital, Navy Medical University of China during January 2016 and December 2018 were retrospectively collected in this study. All surgeries were performed by four professors with equivalent experiences in the surgical treatment of gastric cancer. The exclusion criteria were: ASA-IV status, Synchronous malignant diseases, combined surgery and missing data for estimation of propensity score (Fig. 1).

Through a consensus meeting involving surgeons and biostatisticians, 15 preoperative variables possibly influencing the choice of surgical approach and associated with outcome were identified to enable strict PSM. Each patient’ s propensity score was calculated using a logistic regression model based on age, sex, area, education, body mass index (BMI), operation year, ASA score, operation history, comorbidity, preoperative chemotherapy, preoperatively measured tumor size, clinical T-stage, clinical N-stage, laparoscopic-assisted or open gastrectomy. Patients in iso-URYA and anti-URYA groups were matched 1:1 using the nearest propensity score.

The outcomes included complications, changes in nutritional status, endoscopic findings and gastrointestinal quality of life index (GQLI) [9]. The complication was evaluated by Clavien-Dindo classification [10]. The change in nutritional status was evaluated by the relative values of body weight, hemoglobin, and albumin to the preoperative levels one year after surgery. The endoscopic findings 1 year after surgery were evaluated by the endoscopic ‘residue, gastritis, bile’ (RGB) classification proposed by Kubo [11], higher scores meant worse signs in the remnant stomach.

### Digestive reconstruction procedure

**Fig. 2:**
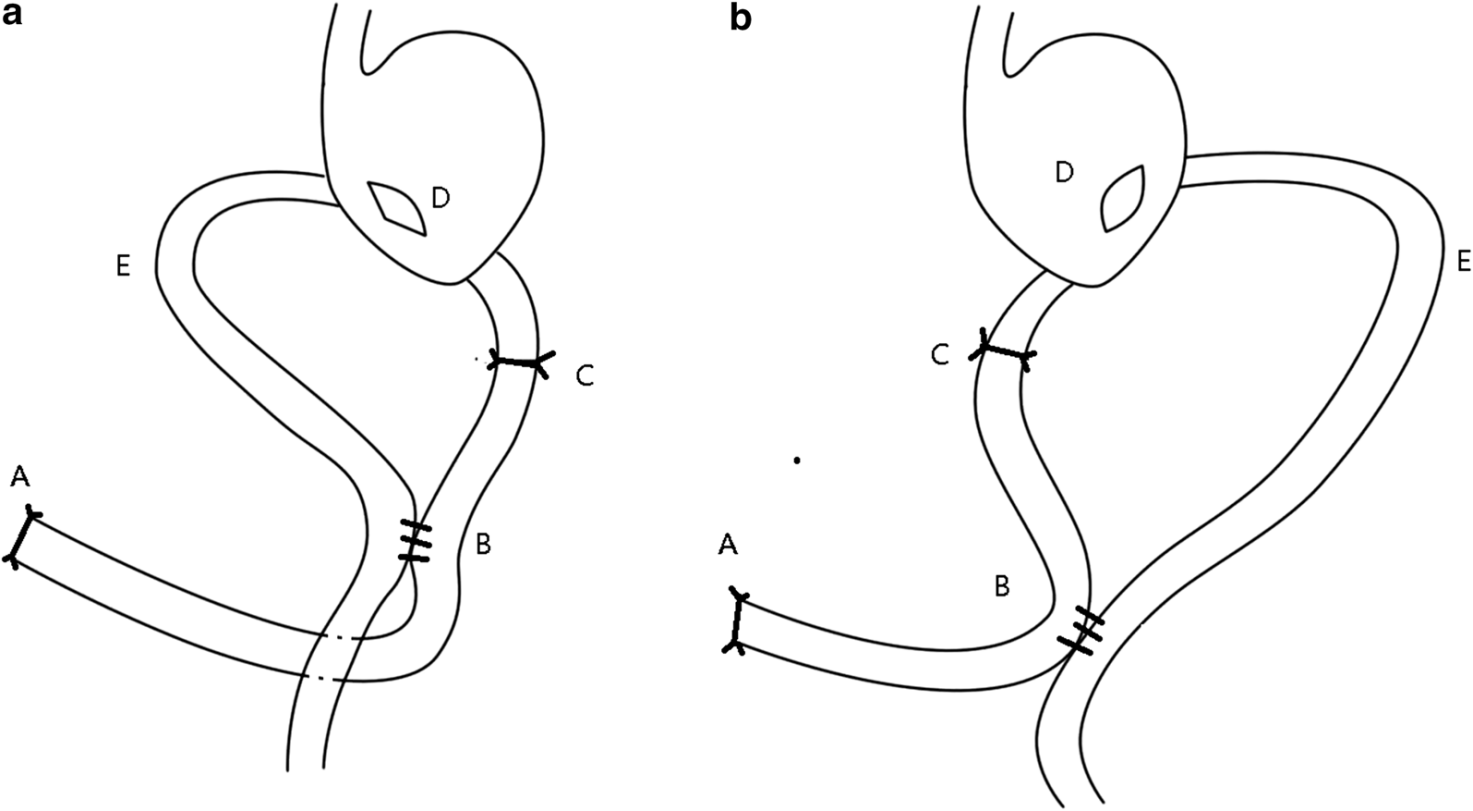
Schematic diagram of uncut Roux-en-y anastomosis (URYA). URYA includes an end-to-side gastrojejunostomy which was constructed approximately 20 cm distal to Treitz ligament and a “Braun enteroenterostomy” which was performed by joining the afferent to efferent limb about 10 and 35 cm away from gastrointestinal anastomosis, respectively, and then the afferent limb was occluded 3 cm away from gastrointestinal anastomosis. **a** Represented iso-URYA and **b** represented anti-URYA. For both **a**, **b**, A represented duodenal stump; B represented Braun enteroenterostomy; C represented afferent occlusion; D represented gastrointestinal anastomosis; E represented efferent loop

After DG with D2 lymphadenectomy, which was following the Japanese gastric cancer treatment guidelines 2014 (ver. 4) [12], the duodenum was transected about 2 cm distal from the pylorus and the stomach was transected about 4–5 cm proximal to the tumor. A small entry was made at the jejunum on the antimesenteric border 20 cm distal to Treitz ligament. Another entry was made at the greater curvature side of posterior wall of gastric stump and 2 cm proximal to the stapling line of remnant stomach. The afferent Loop to lesser curvature for anti-URYA or the afferent loop to greater curvature for iso-URYA side-to-side gastrojejunostomy was performed using a 60-mm linear stapler with a blue cartridge. The “Braun enteroenterostomy” was performed by joining the afferent to efferent limb about 10 and 35 cm away from gastrointestinal anastomosis, respectively. The afferent intestine was blocked by ligation or stapler at about 3 cm away from gastrointestinal anastomosis and several interrupted seromuscular sutures was performed over the blocked site for permanent serosa-to-serosa adhesion (Fig. 2). All patients received antecolic gastrojejunostomy and remnant stomach was not fixed to transverse colon mesentery.

### Statistical method

The continuous data were expressed as mean and standard error and the categorical data were expressed as numbers and proportions. Student t-test or Mann–Whitney U test was used to analyze the continuous data and the Pearson χ^2^ test or the Fisher’s exact test was used to analyze the differences in the categorical data. All the statistical analysis was two tailed test and P values < 0.05 was considered to be statistically significant. All statistical analyses were performed using SPSS ver. 22 for Windows (SPSS Inc., Chicago, IL, USA).

## Result

### Baseline data

A total of 704 gastric cancer patients underwent DG in the period from January 2016 to December 2018. Of those, 92 patients were excluded due to combined organ resection or missing data. Finally, data of 612 patients were collected with 220 patients in anti-URYA group and 392 patients in iso-URYA group. Propensity score matching was performed with 15 covariates (sex, age, BMI, year of surgery, ASA score, patients’ area and education, history of abdominal surgery, history of diabetes, history of smoking, tumor location, tumor size, preoperative chemotherapy, clinical T and N factor) and 183 patients from each group were matched 1:1 and no significant differences in baseline data were observed between two groups after PSM. The details were shown in Table 1.

**Table 1:** Baseline data before and after propensity score matching

Characteristics	Before matching					After matching				
	ANTI (n = 220)		ISO (n = 392)		P	ANTI (n = 183)		ISO (n = 183)		P
	n	%	n	%		n	%	n	%	
Sex										
Male	139	63.2	243	62.0	0.77	116	63.4	114	62.3	0.829
Female	81	36.8	149	38.0		67	36.6	69	37.7	
Age (mean ± SD, year)	63.3	± 8.6	65.6	± 8.4		64.3	± 8.7	64.9	± 8.5	0.563
BMI (mean ± SD, kg/m^2^)	24.9	± 4.4	25.2	± 4.3		24.5	± 4.3	24.8	± 4.5	0.623
Year of surgery										
2016	43	19.6	118	30.1	0.011	28	15.3	34	18.6	0.326
2017	92	41.8	155	39.5		67	36.6	75	41.0	
2018	85	38.6	119	30.4		88	48.1	74	40.4	
ASA score										
I	70	31.8	90	23.0	0.045	49	26.8	50	27.3	0.992
II	109	49.5	210	53.6		98	53.6	97	53.0	
III	41	18.6	92	23.5		36	19.7	36	19.7	
Area										
Urban	95	43.2	206	52.6	0.026	86	47.0	85	46.4	0.917
Rural	125	56.8	186	47.4		97	53.0	98	53.6	
Education (years)										
< 12	41	18.6	99	25.3	0.061	39	21.3	37	20.2	0.797
≥ 12	179	81.4	293	74.7		144	78.7	146	79.8	
History of abdominal surgery										
Yes	120	54.5	198	50.5	0.338	107	58.5	105	57.4	0.832
No	100	45.5	194	49.5		76	41.5	78	42.6	
History of diabetes										
Yes	83	37.7	132	33.7	0.313	75	41.0	70	38.9	0.686
No	137	62.3	260	66.3		108	59.0	113	61.1	
History of smoking										
Yes	83	37.7	168	42.9	0.216	53	29.0	59	32.2	0.496
No	137	62.3	224	57.1		130	71.0	124	67.8	
Tumor location										
Gastric body	79	35.9	99	25.3	0.020	56	26.8	44	24.0	0.331
Between	96	43.6	197	50.3		92	53.6	97	53.0	
Pyloric canal	45	20.5	96	24.5		35	19.7	42	23.0	
Tumor size (cm)										
< 4	123	57.7	259	66.1	0.040	108	59.0	98	53.6	0.292
≥ 4	97	42.3	133	33.9		75	41.0	85	46.4	
Preoperative chemotherapy										
Yes	21	9.5	22	5.6	0.068	14	92.3	15	92.4	0.847
No	199	90.5	370	94.4		169	7.7	168	7.6	
Clinical T factor										
T1	58	26.4	73	18.6	0.009	41	22.4	42	23.0	0.342
T2	46	20.9	99	25.3		44	24.0	31	16.9	
T3	52	23.6	130	33.2		43	23.5	53	29.0	
T4	64	29.1	90	23.0		55	30.1	57	31.1	
Clinical N factor										
N0	101	45.9	219	55.9	0.018	80	43.7	92	50.3	0.209
N1–3	119	54.1	173	44.1		103	56.3	91	49.7	

*ANTI* antiperistaltic uncut Roux-en-Y anastomosis, *ISO* Isoperistaltic uncut Roux-en-Y anastomosis

### Operating findings and complications

Operative findings, including operating time, blood loss, number of harvested lymph node surgery approach, stapler, postoperative hospital stay, did not differ significantly between two groups (Table 2). The method of occlusion of afferent loop was significantly different between two groups (P = 0.002). There were no significant differences in overall complication rates between two groups. The incidence of anastomotic bleeding was higher in the iso-URYA group, although it was not statistically significant (2.2% in the anti-URYA group vs. 4.4% in the iso-URYA group, P = 0.244).

**Table 2:** Operating finding and complication

	ANTI (n = 183)		ISO (n = 183)		P
	n	%	n	%	
Mean operation time, mean ± SD, min	251.5	± 46.4	263.1	± 46.8	0.735
Mean blood loss, mean ± SD, ml	239.6	± 32.4	222.5	± 35.3	0.492
Transfusion	7	3.8	9	4.9	0.617
Harvested lymph node, median (min, max)	29 (17–54)		31 (16–57)		0.565
R0	176	96.2	174	95.1	0.609
Surgery approach					
LAG	90	49.2	76	41.5	0.142
OG	93	50.8	107	58.5	
Stapler					
Circular	49	26.8	61	33.3	0.171
Linear	134	73.2	122	66.7	
Occlusion of afferent loop					
Ligation	101	55.2	162	88.5	0.001
Stapler	82	44.8	21	11.5	
Pathological stage					
Stage 1a	14	7.7	8	4.4	0.868
Stage 1b	25	13.7	23	12.6	
Stage 2a	35	19.1	36	19.7	
Stage 2b	36	19.7	37	20.2	
Stage 3a	29	15.8	34	18.6	
Stage 3b	30	16.4	28	15.3	
Stage 3c	14	7.7	17	9.3	
Postoperative hospital stay, mean ± SD, days	7.7	± 3.7	8.2	± 4.3	0.312
Complication^a^					
Grade 1	39	37.1	36	34.6	0.792
Grade 2	30	28.6	32	30.8	
Grade 3	27	25.7	25	24.0	
Grade 4	9	8.6	10	9.6	
Grade 5	0	0.0	1	1.0	
Early-phase complication					
Incision infection	9	4.9	6	3.3	0.306
Duodenum stump leak	1	0.5	1	0.5	1.000
Anastomotic leak	1	0.5	0	0.0	1.000
Anastomotic bleeding	4	2.2	8	4.4	0.244
Intra-abdominal bleeding	2	1.1	2	1.1	1.000
Intra-abdominal infection	9	4.9	8	3.8	0.804
Delayed gastric emptying	1	0.5	2	1.1	0.562
Pancreatic fistula	10	5.5	5	2.7	0.187
Late-phase complication					
Bowel obstruction	8	4.4	7	3.8	0.792
Internal hernia	3	1.6	2	1.1	0.652
Anastomotic stricture	0	0.0	0	0.0	1.000
Reoperation	2	1.1	2	1.1	1.000
Mortality in 30 days	0	0.0	1	0.5	1.000
Adjuvant chemotherapy	129	66.1	134	71.6	0.309

^a^Clavien-Dindo classification

### Nutritional status, endoscopic findings and GQLI score

The median follow-up time was 26.4 (13.6–42.3) months for iso-URYA group and 28.4 (14.6–44.7) months for anti-URYA group. Follow-up rate was 83.1% for anti-URYA group and 85.3% for iso-URYA group. The nutritional status evaluated by the relative value of body weight, hemoglobin and albumin was not significantly different between two groups. The GQLI score in iso-URYA group was higher than anti-URYA group, but the difference was not statistically significant (P = 0.104). 79.2% patients in anti-URYA group and 76.0% patients in iso-URYA group underwent endoscopic examination. Time interval between surgery and endoscopic examination was similar between two groups (P = 0.726). Endoscopic evaluation of gastritis showed significantly different between two groups (P = 0.036, 30.4% for anti-URYA and 21.5% for iso-URYA), while other two subitems did not differ significantly (P = 0.432 for residual food and P = 0.068 for bile reflux). (Table 3.)

**Table 3:** Nutritional status, endoscopic findings and GQLI score one year after surgery

	ANTI (n = 183)		ISO (n = 183)		P
	n	%	n	%	
Follow-up rate	152	83.1	156	85.3	
Body weight, mean ± SD^a^	90.7	± 8.4	91.1	± 7.9	0.659
Hemoglobin, mean ± SD^a^	97.2	± 7.5	96.9	± 8.1	0.457
Albumin, mean ± SD^a^	104.3	± 9.5	105.2	± 10.1	0.334
Endoscopic findings	145	79.2	139	76.0	
Interval, month, mean ± SD^b^	16.1	± 2.5	15.6	± 3.3	0.726
Residual food					
Grade 0	114	78.6	99	71.2	0.432
Grade 1	13	9.0	18	12.9	
Grade 2	11	7.6	16	11.5	
Grade 3	7	4.8	6	4.3	
Grade 4	0	0.0	0	0.0	
Gastritis					
Grade 0	101	69.7	109	78.4	0.036
Grade 1	29	20.0	28	20.1	
Grade 2	13	9.0	2	1.4	
Grade 3	1	0.7	0	0	
Grade 4	1	0.7	0	0	
Bile reflux					
Grade 0	101	69.7	110	79.1	0.068
Grade 1	44	30.3	29	20.9	
GQLI score, mean ± SD	108.8	± 18.5	112.5	± 17.2	0.104

*GQLI* gastrointestinal quality of life index

^a^Relative value

^b^Interval between surgery and endoscopic examination

### Recanalization

76 patients (ligation for 39 and stapler for 37) in anti-URYA group and 68 patients (ligation for 47 and stapler for 21) in iso-URYA group underwent upper gastrointestinal contrast X-ray 1 year after surgery. 2.3% recanalization (2/86) was observed when the afferent loop was blocked by ligation while 22.4% (13/58) recanalization happened when the afferent loop was blocked by stapler. There were significant differences in recanalization rates between blocked by ligation and blocked by stapler, but there were no significantly differences in recanalization rates between iso- and anti-URYA group (Table 4).

**Table 4:** Recanalization

Recanalization	ANTI (n = 76)			ISO (n = 68)			P
	Ligation	Stapler	P	Ligation	Stapler	P	
YES	1	8	0.013^a^	1	5	0.009^a^	0.554
NO	38	29		46	16		

^a^Fisher’s exact

## Discussion

This research firstly compared iso- and anti-URYA after distal gastrectomy for gastric cancer. No differences were found between them in terms of short-term complications, nutritional status and quality of life 1 year after surgery. But endoscopic examination showed that anti-URYA group had more severe gastritis (P = 0.036). More than 75% patients (79.2% for anti-URYA and 76.0% for iso-URYA) had an endoscopic review in the similar period after surgery, and the result could be convincing. So the anti-URYA might be closely related to reflux gastritis. But our findings could offer another explanation. The recanalization rate was significantly higher after the luminal occlusion by stapler (Table 4), and the luminal occlusion by stapler had a larger proportion in anti-URYA group than iso-URYA group (44.8% vs 11.5%, Table 2). So the anti-URYA group may have higher recanalization rate and further induce more severe gastritis. No significant differences in recanalization rates between iso- and anti-URYA group (P = 0.554, Table 4) was probably because the proportion of patients who underwent contrast X-ray was insufficient (41.5% for anti-URYA group and 37.2% for iso-URYA group).

Little attention was paid to the orientation of peristalsis in previous studies [13–15]. In our institution, the orientation of peristalsis of URYA is mainly determined by surgeon’s personal preference. Surgeons who support anti-URYA argue that iso-URYA has a relatively limited and fixed space between gastrointestinal anastomosis and transverse mesentery and may further increase the incidence of internal hernia. Surgeons who support iso-URYA argue that anti-URYA transects the short gastric artery and may induce gastric stump ischemia. These arguments may exist but our study found no difference in short-term outcomes between two groups. So far, no RCTs focus on this problem. In this retrospective cohort study, we identified sufficient clinically essential covariates from among preoperative variables to maximize the comparability between iso- and anti-groups as far as possible. This methodology using actual clinical data with strict PSM may compensate for RCTs in the context of rapid developments in surgical treatment [16, 17].

The anastomotic bleeding was more common in iso-URYA comparing with anti-URYA (4.4% vs 2.2%, P = 0.240), presumably because of the higher proportion of circular stapler use in iso-URYA group. Circular stapler has poor hemostatic effects by tissue squeezing and the following anastomotic reinforcement by suture may also increase anastomotic bleeding [18, 19]. An increased incidence of pancreatic fistula in anti-URYA group may be related to higher proportion of LAG (5.5% vs 2.7%, P = 0.187). The possible reasons included intraoperative compression of the pancreas with long straight instruments, an inappropriate dissection plane along the pancreas, or thermal damage to the pancreas by energy devices in LAG [17].

URYA can divert biliary and pancreatic secretions away from the remnant stomach more efficiently by blocking the afferent loop and further prevent inflammation and even carcinogenesis of the remnant stomach and esophagus [3–5, 20, 21]. However, the recanalization of the jejunum after surgery may nullify this benefit. The luminal recanalization is caused by the failure of a fibrous healing process between the approximated mucosal surfaces [6, 22]. In this study, two methods, 0 # non-absorbable suture or no-knife linear cutter, were used to block the afferent loop. Only 2 out of 86 had recanalization for the former. 13 out of 58 had recanalization for the latter, of which 2 used 6-row linear stapler and 11 used 3-row linear stapler. So it seems that non-absorbable suture is more suitable for the luminal occlusion basing on the lower recanalization rate comparing with 3-row linear cutter and lower economic costs comparing with 6-row linear cutter. Our experience is that the ligation should be enough to block but not cut the small bowel. Too loose or too tight ligation both can induce early recanalization.

It cannot be denied that the present study had some important limitations. First, it was still retrospective in nature even after very strict PSM. There is no guarantee that all confounding factors were included in our analyses. RCTs will be necessary to assess the accuracy of this type of study. Second, this was a single center study. Therefore, we should be careful when extrapolating our results to all institutions. Third, this study could not survey postoperative symptoms severity. Assessment of subjective symptoms with a well-designed questionnaire might reveal the differences between two groups on early oral feeding or postprandial discomfort.

## Conclusion

In conclusion, the iso-URYA and anti-URYA group present similar results in short term outcomes. The iso-URYA group had lower rate of severe gastritis comparing with anti-URYA group, and the reason lies in the higher proportion of ligation blocking afferent loop in iso-URYA group which leads to lower recanalization rate.

## Data Availability

The datasets used during the current study are available from the corresponding author on reasonable request.

## Abbreviations

URYA: Uncut Roux-en-y anastomosis
iso-URYA: Isoperistaltic URYA
anti-URYA: Antiperistaltic URYA
DG: Distal gastrectomy
GC: Gastric cancer
PSM: Propensity score matching
BMI: Body mass index
LAG: Laparoscopic assisted gastrectomy
